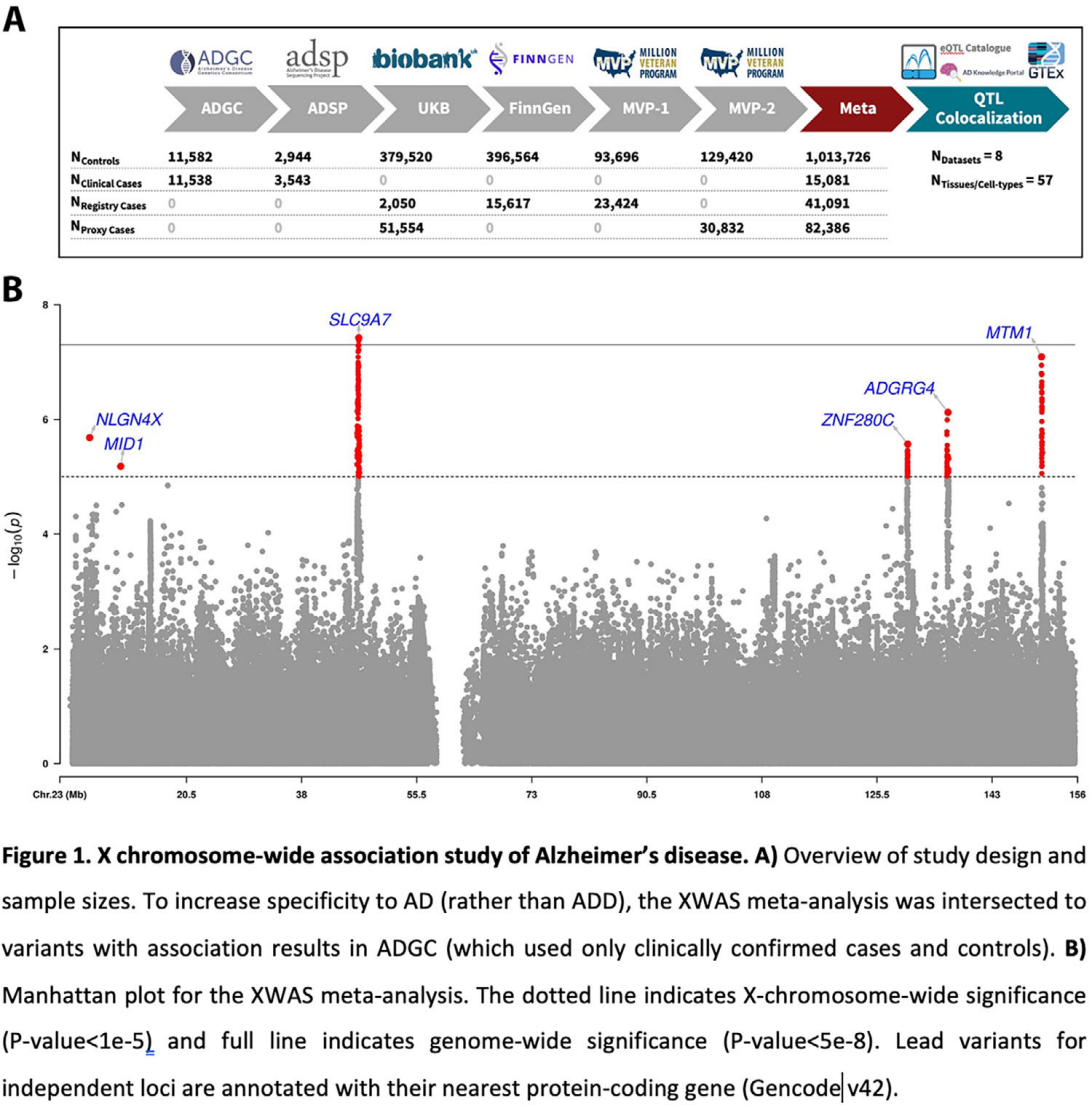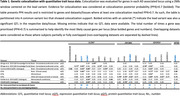# The X chromosome in Alzheimer’s Disease

**DOI:** 10.1002/alz.095315

**Published:** 2025-01-09

**Authors:** Michael E. Belloy, Yann Le Guen, Ilaria Stewart, Joachim Herz, Richard Sherva, Rui Zhang, Victoria Merritt, Matthew S. Panizzon, Richard L. Hauger, J. Michael Gaziano, Mark W. Logue, Valerio Napolioni, Michael D Greicius, the VA Million Veteran Program

**Affiliations:** ^1^ Washington University in Saint Louis, Saint Louis, MO USA; ^2^ Stanford University, School of Medicine, Stanford, CA USA; ^3^ Stanford University School of Medicine, Stanford, CA USA; ^4^ UT Southwestern ‐ Center for Translational Neurodegeneration Research, Dallas, TX USA; ^5^ Department of Medicine (Biomedical Genetics), Boston University Chobanian & Avedisian School of Medicine, Boston, MA USA; ^6^ National Center for PTSD, VA Boston Healthcare System, Boston, MA USA; ^7^ VA San Diego Healthcare System, San Diego, CA USA; ^8^ University of California, San Diego, La Jolla, CA USA; ^9^ Center for Excellence for Stress and Mental Health (CESAMH), VA San Diego Healthcare System, San Diego, CA USA; ^10^ VA Boston Healthcare System, Boston, MA USA; ^11^ Boston University School of Medicine, Boston, MA USA; ^12^ University of Camerino, Camerino Italy

## Abstract

**Background:**

The X‐chromosome remains largely unexplored in Alzheimer’s disease (AD). To address this gap, we performed the first large‐scale X chromosome‐wide association study (XWAS) of AD.

**Method:**

The study overview is shown in **Figure 1A**. We performed a meta‐analysis of XWAS in case‐control, family‐based, population‐based, and longitudinal AD‐related cohorts from the US Alzheimer’s Disease Genetics Consortium (ADGC) and Alzheimer’s Disease Sequencing Project (ADSP), the UK Biobank (UKB), the Finnish health registry (FinnGen), and the US Million Veterans Program (MVP). Genetic data were available from high‐density single‐nucleotide polymorphism (SNP) microarrays and whole‐genome sequencing (WGS). To identify potentially causal genes in associated risk loci, statistical colocalization was evaluated between the local genetic association signal for AD and the genetic association signal for molecular traits such as expression levels of genes within that locus (R‐v.4.2.1, *coloc*). We leveraged public datasets where quantitative trait loci (QTL) for expression and protein levels were available for the X chromosome in brain and non‐brain tissues.

**Result:**

The XWAS included 1,152,284 non‐Hispanic White European ancestry subjects, including 138,558 cases (**Figure 1A**). 6 independent genetic loci passed X‐chromosome‐wide significance (**Figure 1B**), with 4 showing support for causal links between the genetic signal for AD and expression of nearby genes in brain and non‐brain tissues (**Table.1**). One of these 4 loci passed conservative genome‐wide significance, with its lead variant centered on an intron of *SLC9A7* (OR = 1.054, 95%‐CI = [1.035, 1.075]) and colocalization analyses prioritizing both the *SLC9A7* and nearby *CHST7* genes.

**Conclusion:**

We performed the first large‐scale XWAS of AD, which is simultaneously the largest genetic association study of AD to date. We identified several associated loci, with the strongest support for the novel *SLC9A7* locus. *SLC9A7* regulates pH homeostasis in Golgi secretory compartments and is anticipated to have downstream effects on amyloid beta accumulation. Overall, this study significantly advances our knowledge of AD genetics and may provide novel biological drug targets.